# The Natural History and Predictors for Intervention in Patients with Small Renal Mass Undergoing Active Surveillance

**DOI:** 10.1155/2015/692014

**Published:** 2015-04-15

**Authors:** Zaher Bahouth, Sarel Halachmi, Gil Meyer, Ofir Avitan, Boaz Moskovitz, Ofer Nativ

**Affiliations:** ^1^Department of Urology, Bnai Zion Medical Center, 3339414 Haifa, Israel; ^2^Faculty of Medicine, Technion-Israel Institute of Technology, 3200003 Haifa, Israel

## Abstract

*Aim*. To describe the natural history of small renal mass on active surveillance and identify parameters that could help in predicting the need for intervention in patients with small renal masses undergoing active surveillance. We also discuss the need for renal biopsy in the management of these patients. *Methods*. A retrospective analysis of 78 renal masses ≤4 cm diagnosed at our Urology Department at Bnai Zion Medical Center between September 2003 and March 2012. *Results*. Seventy patients with 78 small renal masses were analyzed. The mean age at diagnosis was 68 years (47–89). The mean follow-up period was 34 months (12–112). In 54 of 78 masses there was a growth of at least 2 mm between imaging on last available follow-up and diagnosis. Eight of the 54 (15%) masses which grew in size underwent a nephron-sparing surgery, of which two were oncocytomas and six were renal cell carcinoma. Growth rate and mass diameter on diagnosis were significantly greater in the group of patients who underwent a surgery. *Conclusions*. Small renal masses might eventually be managed by active surveillance without compromising survival or surgical approach. All masses that were eventually excised underwent a nephron-sparing surgery. None of the patients developed metastases.

## 1. Introduction

Renal cell carcinoma (RCC) represents 2-3% of all cancers, with an age-adjusted incidence of 5.8 per 100,000 in developed countries [1]. During the last two decades, there has been an annual increase of about 2% in the incidence of RCC worldwide [2]. RCC is the most common solid lesion in the kidney and accounts for 90% of all kidney malignancies. Small renal mass (SRM) is defined as solid enhancing tumors up to 4 cm in maximal diameter and accounts for up to 66% of all renal tumors [3], most of them being diagnosed incidentally.

At least 20% of SRMs presumed to be RCC are in fact benign masses when biopsied or excised [4]. In a retrospective study of 2770 patients with renal masses, Frank et al. showed a direct correlation between tumor size and probability of malignancy, with an increase of 17% for each growth of 1 cm in size of mass [5]. Most SRMs show a slow growth rate and rarely progress to metastatic disease [6]. Because of the slow growth rate and the low risk of metastases, active surveillance with delayed treatment for patients showing progression can be considered in patients with a newly diagnosed SRM [7], especially those with significant comorbidities, but it could also be acceptable regardless of the patient age in very small renal masses (<1 cm) as suggested by Gill et al. [8]. However, indications for active treatment and predictors for progression are not well defined.

In addition, other treatment options exist for small masses especially for patients who do not fit for surgery or prefer a less radical treatment. Such treatment options include renal cryoablation and radiofrequency ablation.

Even though NSS is considered the gold-standard in managing small renal masses, it has been shown that surgery could adversely affect the renal function, adversely affecting survival [9], although far less than radical nephrectomy.

The aim of the current study was to assess the results of active surveillance for SRMs and to define parameters that could differentiate those who need surgery from those that can be managed by surveillance only. We also discuss the need for biopsy in these patients.

## 2. Materials and Methods

We retrospectively analyzed the radiological and clinical data collected from a total of 101 contrast-enhancing SRMs diagnosed between September 2003 and January 2013 at our Urology Department at Bnai Zion Medical Center. The inclusion criteria for surveillance were contrast-enhancing renal mass of 4 cm or less in maximal diameter, risk factors for end-stage renal disease, multiple major comorbidities, and patient preference. Risk factors for end-stage renal failure (e.g., impaired baseline renal function, poorly controlled hypertension, and long-standing or poorly controlled diabetes) were included because of the risk of subsequent decrease in renal function and the need for renal replacement therapy following renal surgery. Exclusion criteria included patients who did not have a follow-up of at least 12 months (*n* = 23). We ended up with a total of 70 patients with 78 masses clinically staged as T1aN0M0. The following data was obtained from the medical records: age, gender, comorbidities, history of renal and nonrenal malignancies, laterality, number and size of the lesions, date and modality of follow-up imaging, and all relevant information concerning surgery when it was done. Follow-up protocol included reimaging every six months in the first year by CT-Urography or MR-Urography in order to determine any change in the lesion size and assess disease progression. Starting in the second year, follow-up protocol included an alternating CT-Urography or MR-Urography (in patients with impaired renal function) and sonography every six months. Masses measurement was done by one radiologist who was not aware of patients' outcome and was based on the measurement of the maximal tumor diameter. Indications for intervention were high growth rate, a lesion that grew beyond 40 mm, and patient or doctor preference.

*Statistical Analysis*. The mean tumor growth rate was calculated by the absolute change in maximal diameter in imaging at last available follow-up and at diagnosis and stated as cm/year. Categorical data were analyzed using Fisher's exact test and Chi-square test. Continuous variables were analyzed using *t*-test. Significance level was set as *P* value < 0.05. A multivariate analysis was done using a stepwise logistic regression and included the following parameters: sex, age, laterality, size of mass on diagnosis, previous NSS, history of cancer, and growth rate. For statistical analysis, MedCalc version 12.5 was used.

## 3. Results

Among the 70 patients, 39 were males and 31 were females. Mean patients age was 68 years (range 47–89 years). Mean Charlson Comorbidity Index was 4.5, with minority of patients having a score of less than 2. Fifty-six percent of the masses were in men. Six of 70 patients had multiple SRMs; of them three had bilateral synchronous lesions. One patient had SRM in a single kidney (first kidney removed of RCC) and 6 patients had previously undergone renal surgery, one for oncocytoma and five for RCC. Mean follow-up time was 34 months (range 12–112 months). Patients and masses characteristics are summarized in Table 1.

Seven patients (10%) with 8 SRMs underwent treatment, all of them by NSS. All patients were referred to NSS as it is considered the gold-standard treatment and because of limited data and experience in other treatment options (e.g., ablative therapies). On histopathology, six of the excised masses were RCC and two oncocytomas (Table 2). None of the surgically treated patients had recurrence during the study follow-up.

The mean growth rate of all SRMs in our study was 0.17 cm/year (range −0.29 to 0.88 cm/year). Fifty-four of the 78 masses (69%) showed growth in size of at least 2 mm between diagnosis and last available imaging study. All other 24 masses (31%) did not show any growth during follow-up period. Among the 54 masses that increased in size, the mean growth rate was 0.25 cm/year.  Table 3 shows the growth rate of SRMs as seen in our study compared to previous studies.

Three patients from the surveillance group had indications for intervention as detailed before, but none of them underwent such a surgery, one because of benign histology on biopsy and two because of very advanced age.

**Figure 1:**
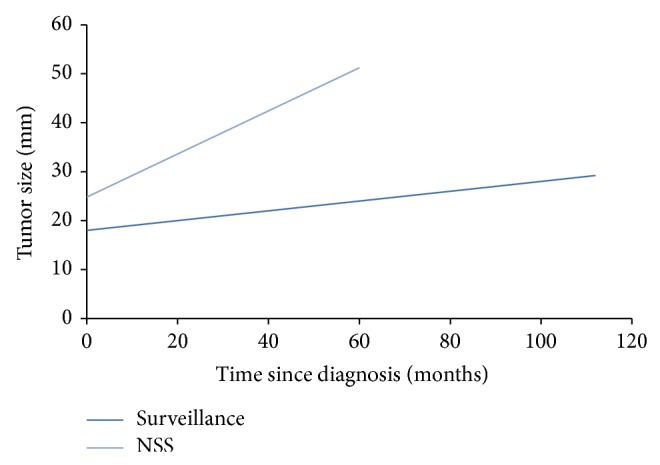
Growth rate of small renal masses. Growth rate of masses that underwent active treatment was 0.53 cm/year (light line) which was significantly higher than that observed for masses which were managed expectantly, 0.12 cm/year (dark line). *P* < 0.0001.

We did a univariate and multivariate analysis to check for differences in patients' parameters in both groups. Univariate analysis showed size at diagnosis and tumor growth rate as the only parameters with significant difference. The mean growth rate of the lesions that were actively treated was 0.53 cm/year (95% CI 0.33, 0.71) as opposed to 0.12 cm/year (95% CI 0.08, 0.17) for masses managed expectantly (*P* < 0.0001) (Figure 1).

**Figure 2:**
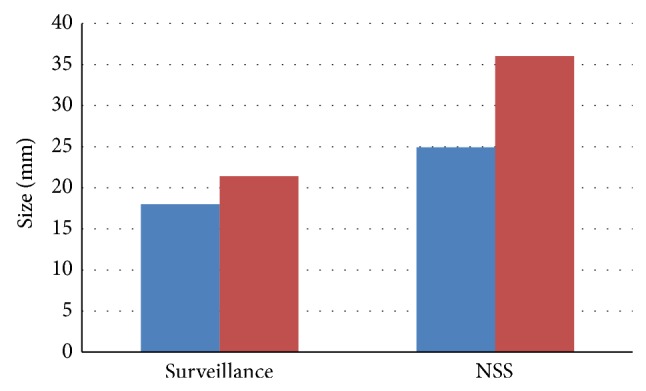
Masses size in both groups as observed at diagnosis and on last follow-up. Blue demonstrates size at diagnosis and red demonstrates size at last available follow-up. Masses diameter at diagnosis was significantly (*P* = 0.04) smaller in the group of patients who were managed conservatively (18 mm versus 24.9 mm).

Mean tumor diameter at diagnosis for the entire group was 18.7 mm (range 5–40 mm). Patients who remained on active surveillance had significantly smaller masses at diagnosis as compared to patients who underwent surgery, 18 mm (95% CI 15.9, 20.2) and 24.9 mm (95% CI 16.7,33), respectively, *P* = 0.04. The mean tumor diameter on last available follow-up was 21.4 mm (range 7–40) in the group of patients who remained on active surveillance and 36 mm (range 19–59) in patients who underwent a NSS (Figure 2).

In multivariate analysis the only parameter with significant difference between the intervention group and the follow-up was tumor growth rate.

## 4. Discussion

Our data demonstrated that a significant number (31%) of SRMs did not grow at all, while many (59%) demonstrated a slow growth rate (0.25 cm/year). Only 8 masses in 7 patients (10%) underwent an intervention, in which the growth rate was significantly higher (4.5-fold increase, *P* < 0.0001).

Similar data was reported by Jewett et al. showing that 37% of SRMs did not grow [12]. Mason et al. reported 15% of masses not growing in size [13], in a cohort of 84 masses with a mean size of 2.3 cm at diagnosis. The mean growth rate of the 54 masses that increased in size was 0.25 cm/year in our study. This rate is similar to other previous studies [12, 13].

Follow-up revealed stage progression in only 3 patients (4%) who increased from clinical stage T1a to T1b. None of the patients showed progression to clinical stage T ≥ 2 nor to metastatic disease. No functional deterioration among the study participants was observed, and no patient required a renal replacement therapy. Such limited stage progression was also reported in previous studies [13].

Our initial conservative treatment approach, that is, active surveillance, did not compromise our ability to carry out a nephron-sparing surgery in patients mandated to active treatment, which is the gold-standard surgery. Moreover, postoperative follow-up of the 7 patients who were operated on did not demonstrate locoregional or metastatic progression. Several studies reported favorable outcomes for patients with SRMs who were managed conservatively, with only sporadic cases of stage progression [13, 15].

Tumor size at diagnosis was significantly higher (24.9 mm versus 18 mm, *P* = 0.04) in masses that were eventually subjected to surgical intervention. Similarly, Mason et al. demonstrated that larger tumors (≥24.5 mm) have a higher growth rate leading to higher rate of intervention [13]. We saw a similar trend in the current study, with a growth rate of 0.23 cm/year for masses ≥24.5 mm and 0.14 cm/year for masses <24.5 mm, but without statistical significance (*P* = 0.17). This fact shows that the size of tumor at diagnosis could be helpful in selecting the optimal management, as larger masses tend to grow faster than smaller ones.

In our study, we did not require a pretreatment biopsy for enrollment. Among our patients, only three underwent a renal biopsy, all of them because of personal preference. One patient was an 80-year-old patient, with a mass of 18 mm at diagnosis, who remained under follow-up for additional 46 months without progression, and his biopsy showed G1 clear-cell RCC. The second patient was diagnosed with a 25 mm mass, whose biopsy showed G2 clear-cell RCC and underwent a surgery when the mass was 43 mm, 4 years after initial diagnosis. The last patient was from the surveillance group who had a mass of 40 mm who underwent a biopsy that showed a benign lesion.

It should be mentioned that two of the eight SRMs that were excised were oncocytoma. One could argue that a biopsy could have changed the management by omitting surgical treatment. However, even in the presence of oncocytic cells in the biopsy specimen, the possibility of hybrid tumor cannot be ruled out. The issue of a pretreatment biopsy is still under debate and requires further studies. Other reasons to omit biopsy include nondiagnostic results in as high as 20% in some studies, the need for a repeat biopsy in 3%, and grade 1 and grade 3 complications in 10.1% and 0.3%, respectively [16]. However, more recent studies suggest better results and less nondiagnostic biopsies [17].

Taken together, the results of the current study and of previously published studies indicate that most SRMs possess a favorable biology. Conservative treatment approach is safe and effective, and if a surgery is needed during the follow-up, a NSS could still be performed.

In our experience, the most significant predictors for intervention were tumor growth rate and tumor size at diagnosis. Larger tumors and high growth rate should mandate an early intervention rather than active surveillance because of the higher tendency of being operated on.

Eventually, the major limitation of our study was the fact that it was retrospective. Another limitation is the relatively small number of patients. One more limitation is the relatively short mean follow-up. A prospective study with a larger number should be done in order to validate our results and to better define thresholds for initiating treatment.

## 5. Conclusions

Active surveillance with serial imaging studies is a reasonable and safe management option for newly diagnosed SRMs. A small percentage of these masses require intervention and delayed treatment with NSS can still be carried out although the masses grew in size. Moreover, delayed treatment does not compromise overall long-term outcomes.

## Figures and Tables

**Table 1:** Patients and masses characteristics with statistical significance.

Feature	Active surveillance	NSS	*P* value
Age			0.88
Mean (range)	68.6 (49–89)	69 (47–87)	
Sex			0.63
Men	34	5	
Women	29	2	
Side			0.19
Right	30	6	
Left	40	2	
Follow-up, months	34.9 (12–112)	25.9 (13–46)	0.28
Size at Dx, mm	18 (5–40)	24.9 (8–40)	**0.04**
Growth rate, cm/year	0.12 (−0.29–0.65)	0.53 (0.18–0.88)	**<0.0001**

**Table 2:** Characteristics of patients who underwent NSS.

Number	Age	Sex	Size at Dx (mm)	Size on Sx (mm)	Growth rate (cm/year)	Time to surgery (months)	Histopathology	Mass
1	77	M	30	45	0.47	38	Oncocytoma	Exophytic
2	57	F	40	59	0.88	26	Oncocytoma	Exophytic
3	54	M	31	37	0.31	23	Pap RCC	Exophytic
4	47	F	8	20	0.46	32	CC RCC, G2	Hilar
5	82	M	28	37	0.67	16	CC RCC, G3	Exophytic
6	87	M	25	43	0.47	46	CC RCC, G3	Exophytic
7a	75	M	20	28	0.74	13	CC RCC, G2	Endophytic
7b	75	M	17	19	0.18	13	Pap RCC	Exophytic
Average	**69**		**24.8**	**36**	**0.53**	**25.9**		

Pap RCC: papillary renal cell carcinoma; CC RCC: clear-cell type renal cell carcinoma; G = Fuhrman grade; 7a and 7b represent two lesions in the same patient; lesion 7b was excised because the patient was undergoing an excision for lesion 7a; otherwise it could still be on active surveillance. Mean growth rate without lesion 7b is 0.57 cm/year compared to 0.53 cm/year with lesion 7b.

**Table 3:** Growth rate of SRMs as seen in several studies.

Study	Year	Number of lesions	Number of patients	Mean mass size (cm)	Mean growth rate (cm/year)	Mean follow-up (months)
Chawla et al. [10]^‡^	2006	234	NA	2.6	0.28	34
Kunkle et al. [11]	2007	106	89	2.0^*^	0.19^*^	29^*^
Jewett et al. [12]	2011	151	82	2.1	0.13	28
Mason et al. [13]	2011	84	82	2.3^*^	0.25^*^	36^*^
Smaldone et al. [14]^‡^	2012	284	259	2.3	0.31	33.5

Current study	2015	78	70	1.9	0.17	34

NA: not available. ^*^Median. ^‡^Review article.
